# Physical Health Problems in Psychosis: Is It Time to Consider the Views of Family Carers?

**DOI:** 10.3389/fpsyt.2018.00668

**Published:** 2018-12-06

**Authors:** Juliana Onwumere, David Shiers, Fiona Gaughran

**Affiliations:** ^1^Department of Psychology, Institute of Psychiatry, Psychology and Neuroscience, King's College London, London, United Kingdom; ^2^South London and Maudsley NHS Foundation Trust, Bethlem Royal Hospital, Beckenham, United Kingdom; ^3^Division of Psychology and Mental Health, University of Manchester, Manchester, United Kingdom; ^4^Psychosis Research Unit, Greater Manchester Mental Health NHS Trust, Manchester, United Kingdom; ^5^Department of Psychosis Studies, Institute of Psychiatry, Psychology and Neuroscience, King's College London, London, United Kingdom

**Keywords:** carers, families, psychosis, severe mental illness, physical health

## Introduction

People living with a diagnosis of psychosis have depleted social networks (1); a picture which is already evident at the first episode stage (2). The development of any psychotic illness (e.g., schizophrenia) will significantly and adversely impact multiple areas of a person's life and functioning, and leave many in socially marginalized positions, excluded from peer groups, and educational and employment opportunities (3, 4).

The vast majority of people living with psychosis reside in community based settings and receive the bulk of their care and support from informal carers, who are typically close relatives (e.g., parents, partners, children, siblings). Informal carers have long been recognized for the vital role they play in supporting patients with psychosis to recover, and their role in improving a broad range of outcomes (5). Such outcomes include reducing rates of relapse and need for inpatient care (6), and improving engagement in and outcomes from prescribed treatments (7, 8). For many, the time spent in caregiving duties can often exceed those recorded for a full-time job (9). Family carers are often the first to observe signs of deterioration in their relative's psychiatric health (10) and play an instrumental role in mobilizing appropriate service responses and treatments (11). Interestingly, recent developments in the literature have also highlighted that life expectancy in patients is significantly elevated in those with carer support compared to those without (12, 13). Reininghaus and colleagues, for example, completed a 10 years follow up of first episode psychosis cases in England. Their results suggested that patients with families were 90% more likely to be alive at follow up (i.e., less likely to have experienced a death attributable to unnatural cause e.g., suicide), compared to peers without no documented family support (12). Ran et al. (13) who assessed families living in China observed that over a 14 years follow up period, it was patients with family support who had improved survival rates at 70.9% compared to rates of 47.5% for those without support.

## Caregiving and Patient Physical Health

There is an extensive body of literature documenting elevated rates of physical health comorbidity in people with psychosis and other severe mental illnesses (SMI) (14–16), and their significantly reduced life expectancy rates compared to the general population (17–19). This gap is reported to be increasing (20) with some studies reporting a range between 10 and 30 years (14).

Though rates of deliberate self-harm and suicide are elevated in SMI (21); for the majority of people, the excess mortality rates are attributable to avoidable and treatable conditions that have modifiable lifestyle risk factors (e.g., sedentariness, tobacco use) (22–24), and inequality in access to healthcare and treatment (25). However, despite the role played by family carers in helping to optimize recovery outcomes for patients, carer experiences of patient physical health comorbidity in psychosis and other SMI have been largely overlooked by researchers and clinicians alike. To date, there have been only a handful of studies that have purposively sought the perspective of carers on matters related to patient physical health (26–29). Findings from our group (26), which were based on a combination of individual and group interviews with carers of adults with long-term psychosis, highlighted carers' exposure to a broad range of physical health conditions in patients. These included cancer and sexual health problems, alongside more frequently reported conditions of poor diet/high body mass index, diabetes, smoking and respiratory related concerns, and poor oral health. Five key themes capturing carers' experience of physical problems in patient groups were extracted from interviews. The themes focused on carers' perception and subjective experience of gaps in service provision for relatives with psychosis and concomitant physical health conditions, and the carer's role in meeting unmet service needs, including going to fitness classes with their relative. The identified themes also centered on the difficult conversations carers often found themselves having with their relative on physical health matters such as smoking cessation, dietary choices/weight management, seeing a general practitioner, how such conversations affected the quality of their caregiving relationship, and the consideration and forethought that preceded decisions to facilitate discussions. The impact of patient physical health on carers' own health and well-being were also noted as an important theme (26). Similar work from Happell and colleagues in Australia (27), as part of their qualitative investigations with a broad range of mental health carers, also highlighted the impact of patient physical health on carer health status. Their findings also identified a theme that reflected carers' beliefs about the fusion of mental and physical health problems. For example, it included concerns of how medication side-effects led to physical health problems (e.g., weight gain) which, in turn, impacted their mental health and ability to engage in strategies to improve their physical health. Lawn et al. (28) interviewed 12 family carers on their experiences of patient smoking behaviors. The results illustrated carers' struggles and perceived responsibilities for managing patient physical health, their own accommodation and facilitation of patient smoking, and the dissonance they experienced over their accommodation behaviors and their understanding of the negative health implications of smoking.

Though there will be overlapping features with other SMI conditions (e.g., BPAD), and with physical health problems that occur independent of co morbid severe mental health problems, the unique presentation of psychosis in an individual which, for many, can include the experience of paranoia, suspiciousness, hallucinations, and affective and cognitive disturbance, will impact their ability to communicate clearly and effectively with others. For example, hallucinatory experiences and paranoia can render it difficult for patients to first identify problems, articulate concerns or changes in their physical health and/or attribute changes in their functioning to possible physical health factors. Likewise, their style and patterns of communication (e.g., thought disorder) might increase the likelihood that others might misunderstand their communications. Patient symptomatology can also interfere with a carer's efforts and confidence to approach physical health issues in relatives through underlying concern and experience that their communications, behaviors, and intentions might be misunderstood and/or cause upset. Carers can find themselves feeling individually responsible for policing or modifying patient unhealthy behaviors and also accommodating behaviors to maintain calm and equilibrium (28). Given the complexity of patient symptoms, some carers might consider that in the “grand scheme” of things, trying to address the physical health issues faced by their relative are secondary to meeting the immediate challenges presented by their mental health problems [e.g., (9, 30)]. For example, if their relative is socially isolated with minimal activities and feelings of joy during the day, then encouraging their relative to refrain from consuming a favorite, yet calorific/low nutritional value, food item or smoking a cigarette can seem difficult to implement as it might mean their relative is left with no positive experiences during the day. It is these additional layers of complexity within the caregiver role and caregiving relationship that have been overlooked within the literature and, consequently, carers have been left to navigate this difficult terrain themselves without support and evidence driven guidance.

## Carer and Patient Health

In comparison to the general population, poorer health status, including sleep disturbance, are also elevated in carers of patients with SMI (31–34), and patient physical and mental health problems have been linked to carer's poorer physical health (9, 26–28). Perlick et al. (32) reviewed the health status of 264 carers of people living with bipolar and psychosis spectrum disorders. The results highlighted that, in the preceding 5 years, approximately two thirds of carer participants reported experiencing health conditions such as hypertension and diabetes, and one third experienced at least two serious physical health conditions. The need for clinicians and researchers to extend discussions and service initiatives on patient physical health to incorporate carer needs are indicated. More recently, Poon et al. (33) assessed the mental and physical health status of 42 carers of patients with first-episode psychosis in Australia. Approximately one quarter of carers had high risk for Type 2 diabetes, one third had hypertension and just under 80% were overweight.

## Recommendations for Improving Physical Health Outcomes in SMI: Supporting the Role of Carers

In the United Kingdom, current treatment guidance recommends regular physical health monitoring in people with SMI (35, 36). However, monitoring rates can be variable (37) and the role played by carers in supporting these guidelines is unknown. Strategies to improve patient physical health have failed to acknowledge an ever expanding role carers adopt in supporting relatives, directly and indirectly, with their physical health (e.g., remembering appointments, escorting relatives to appointments, rebooking missed appointments, paying for gym membership). In light of emerging evidence, we argue that discussions on optimizing patient physical health and ensuring parity of esteem in physical and mental health provision must consider the role of carers, the impact of patient health on carer health and well-being and the caregiving relationship, and to consider what roles, (if any), carers might want in supporting improved physical health outcomes in patients.

The available literature suggests that mental health carers can all too often feel marginalized by service providers (38, 39) despite their reported need to be more involved and treated as partners in patient care, where their unique roles and expertise are recognized (40, 41). Though there are no specific guidelines on how best to support carers to address and cope with the impact of physical health comorbidity in SMI, the literature does offer some helpful indications. We already know that carers report a lack of information about physical health issues in psychosis and welcome opportunities to improve their understanding (26). It is conceivable, therefore, that carers might benefit from:

a. recognition of their caring role, knowledge and expertise about the patient, and their particular needs for information and support regarding physical health comorbidity,
b. psychoeducation interventions, from first onset, about what constitutes good physical health and identifying factors that help to encourage good health,
c. specific guidance on facilitating positive styles of communication on sensitive health issues (e.g., weight),
d. guidance on strategies (i.e., small, feasible) that could be used to promote improved patient health status. The strategies should be tailored toward the needs of a caregiving relationship and consider how psychosis can affect patient perceptions of events, people, and communication,
e. greater awareness of the research underpinning the global focus on decreasing physical health morbidity in SMI. This should be delivered with a balanced message of hopefulness and the potential for positive change and improvement avoiding the negative impacts of research and health messages focused exclusively on excess mortality and poorer outcomes.

Finally, there is an established evidence base detailing the broader impact of patient mental health problems on carer functioning such as burden (34, 42). However, a more nuanced understanding of how patient physical health problems interact with and impact carer health would seem a fruitful direction to follow [e.g., (31–34)]. Strategies to ensure carers have opportunities to reflect on the subjective impact of patient multi-morbidity on their own well-being and to access support in identifying adaptive coping and health management strategies (e.g. consulting with their own primary care physician, stress management, lifestyle interventions to maintain their own physical health) could be beneficial (43). These recommendations remain consistent with treatment and best practice guidance for providing carer focused interventions in SMI (35).

## Conclusion

Informal carers are providing the bulk of care for people living with psychosis and many will reside in the same household and are therefore important figures in a patient's social network. Patient outcomes, including life expectancy, are enhanced where there is carer support and involvement, and overall care costs are reduced. There are sound clinical and economic arguments for the informal carer (family) perspective to be sought on discussions and strategies to develop and implement lifestyle interventions for improved patient physical health in SMI. These arguments remain strong given that carers are uniquely placed in the patient's immediate environment to impact change in patient environment and lifestyles. Carers want holistic and integrated approaches to patient care that are robust enough to take account of complex comorbid clinical presentations, and for this to occur irrespective of whether patients access mental or physical health services. Patient well-being, including optimal levels of physical health as part of that, are outcomes of interest and importance for carers. We have reached a stage where a more detailed understanding of carer needs, specifically as they relate to physical comorbidities in patients and their contributions to improving outcomes, are required.

## Author Contributions

JO prepared the first draft of the manuscript. DS and FG contributed to manuscript revision and approved the submitted version.

### Conflict of Interest Statement

JO has received teaching funding from Otsuka Lundbeck Alliance. FG has received support or honoraria for CME, advisory work and lectures from Lundbeck, Otsuka and Sunovion, and has a family member with professional links to Lilly and GSK, including shares. FG is in part funded by the National Institute for Health Research Collaboration for Leadership in Applied Health Research and Care Funding scheme, by the Maudsley Charity and by the Stanley Medical Research Institute. DS is expert advisor to the NICE centre for guidelines and a member of the current NICE guideline development group for Rehabilitation in adults with complex psychosis and related severe mental health conditions; Board member of the National Collaborating Centre for Mental Health (NCCMH); views are the authors and not those of NICE or NCCMH. All authors declare that the research was conducted in the absence of any commercial or financial relationships that could be construed as a potential conflict of interest.

## References

[1] Palumbo C Volpe U Matanov A Priebe S Giacco D. Social networks of patients with psychosis: a systematic review. BMC Research Notes (2015) 8:560. 10.1186/s13104-015-1528-7 26459046 PMC4603917

[2] Sündermann O Onwumere J Kane F Morgan C Kuipers E. Social networks and support in first episode psychosis: exploring the role of loneliness and anxiety. Soc Psychiatry Psychiatr Epidemiol. (2014) 49:359–66. 10.1007/s00127-013-0754-3 23955376 PMC4081600

[3] Henry LP Amminger GP Harris MG Yuen HP Harrigan SM Prosser AL et al. The EPPIC follow-up study of first-episode psychosis: longer-term clinical and functional outcome 7 years after index admission. J Clin Psychiatry (2010) 71:716–28. 10.4088/JCP.08m04846yel 20573330

[4] Marwaha S Johnson S. Schizophrenia and employment: a review. Soc Psychiatry Psychiatr Epidemiol. (2004) 39:337–49. 10.1007/s00127-004-0762-4 15133589

[5] The Schizophrenia Commission The Abandoned Illness: A Report from the Schizophrenia Commission. London: Rethink Mental Illness (2012).

[6] Norman RMG Malla AK Manchanda R Harricharan R Takhar J Northcott S. Social support and three-year symptom and admission outcomes for first episode psychosis. Schizophr Res. (2005) 80:227–34. 10.1016/j.schres.2005.05.006 15964175

[7] Doyle R Turner N Fanning F Brennan D Renwick L Lawlor E. First-episode psychosis and disengagement from treatment: a systematic review. Psychiatr Serv. (2014) 65:603–11. 10.1176/appi.ps.201200570 24535333

[8] Garety PA Fowler DG Freeman D Bebbington P Dunn G Kuipers E. Cognitive behavioural therapy and family intervention for relapse prevention and symptom reduction in psychosis: randomised controlled trial. Br J Psychiatry (2008) 192:412–23. 10.1192/bjp.bp.107.043570 18515890

[9] Brain C Kymes S Di Benedetti DB Brevig T Velligan DL. Experiences, attitudes and perceptions of caregivers of individuals with treatment resistant schizophrenia: a qualitive study. BMC Psychiatry (2018) 18:253. 10.1186/s12888-018-1833-5 30103719 PMC6090592

[10] Birchwood M. Early intervention in schizophrenia: theoretical background and clinical strategies. Br J Clin Psychol. (1992) 31:257–78. 10.1111/j.2044-8260.1992.tb00994.x 1393156

[11] Connor C Greenfield S Lester H Channa S Palmer C Barker C. Seeking help for first episode psychosis: a family narrative. Early Interv Psychiatry (2016) 10:334–45. 10.1111/eip.12177 25303624

[12] Reininghaus U Dutta R Dazzan P Doody GA Fearon P Lappin J. Mortality in schizophrenia and other psychoses: A 10-year follow-up of the ?SOP first-episode cohort. Schizophr Bull. 41:664–73. 10.1093/schbul/sbu138 25262443 PMC4393685

[13] Ran M Chui CHK Wong IY Mao W Lin F Lui B & Chan CL. Family caregivers and outcome of people with schizophrenia in rural China: 14-year follow-up study. Soc Psychiatry Psychiatr Epidemiol. (2016) 51:513–20. 10.1007/s00127-015-1169-0 26724945

[14] De Hert M Cohen D Bobes J Cetkovich-Bakmas M Leucht S Ndetei DM Physical illness in patients with severe mental disorders. II. Barriers to care, monitoring and treatment guidelines, plus recommendations at the system and individual level. World Psychiatry (2011) 10:138–51. 10.1002/j.2051-5545.2011.tb00036.x 21633691 PMC3104888

[15] Vancampfort D Correll CU Galling B Probst M De Hert M Ward PB. Diabetes mellitus in people with schizophrenia, bipolar disorder and major depressive disorder: A systematic review and large scale meta-analysis. World Psychiatry (2016) 15:166–74. 10.1002/wps.20309 27265707 PMC4911762

[16] Ventriglio A Gentile A Stella E Bellomo A. Metabolic issues in patients affected by schizophrenia: Clinical characteristics and medical management. Front Neurosci. (2015) 9:297. 10.3389/fnins.2015.00297 26388714 PMC4558473

[17] Hjorthøj C Stürup AM McGrath JJ Nordent M. Years of potential life lost and life expectancy in schizophrenia: A systematic review and meta-analysis. Lancet Psychiatry (2017) 4:295–301. 10.1016/S2215-0366(17)30078-0 28237639

[18] Walker ER McGee Druss BG (2015). Mortality in mental disorders and global disease burden implications: a systematic review and meta-analysis. JAMA Psychiatry (2015) 72:334–1. 10.1001/jamapsychiatry.2014.2502 25671328 PMC4461039

[19] Chesney E Goodwin GM Fazel S.R Risk of all cause and suicide mortality in mental disorders: a meta-review. World Psychiatry (2014) 13:153–60. 10.1002/wps.20128 24890068 PMC4102288

[20] Nielsen RE Uggerby AS Jensen SO McGrath JJ. Increasing mortality gap for patients diagnosed with schizophrenia over the last three decades–a Danish nationwide study from 1980 to 2010. Schizophr Res. (2013) 146:22–7. 10.1016/j.schres.2013.02.025 23523021

[21] Hor K Taylor M. Suicide and schizophrenia: a systematic review of rates and risk factors. J Psychopharmacol. (2010) 24:81–90. 10.1177/1359786810385490 20923923 PMC2951591

[22] Callaghan RC Veldhuzen S Jeysingh T Orlan C Graham C Kakouris G Patterns of tobacco- related mortality among individuals diagnosed with schizophrenia, bipolar disorder, or depression. J Psychiatr Res. (2014) 48:102–10 10.1016/j.jpsychires.2013.09.014 24139811

[23] Stubbs B Williams J Gaughran F Craig T How sedentary are people with psychosis? A systematic review and meta-analysis. Schizophr Res. 2016:171:103–9. 10.1016/j.schres.2016.01.034 26805414

[24] Moore S Shiers D Daly B Mitchell AJ Gaughran F. Promoting physical health or people with schizophrenia reducing disparities in medical and dental care. Acta Psychiatr Scand. (2015) 132:109–21. 10.1111/acps.12431 25958971

[25] Chadwick A Street C McAndrew S Deacon M Minding our own bodies: reviewing the literature regarding the perception of service users diagnosed with serious mental illness on barriers to accessing physical health care. Int J Ment Health Nurs. (2012) 21:211–9. 10.1111/j.1447-0349.2011.00807.x 22533328

[26] Onwumere J Howes S Shiers D Gaughran F. Physical health problems in people with psychosis: the issue for informal carers. Int J Soc Psychiatry (2018) 64:381–8. 10.1177/0020764018763684 29584519

[27] Happell B Wilson K Platania-Phung C Stanton R. Physical health and mental illness: listening to the voice of carers. J Ment Health (2017) 26:127–33. 10.3109/09638237.2016.1167854 27102585

[28] Lawn S McNaughton D Fuller L What carers of family members with mental illness say, think and do about their relative's smoking and the implications for health promotion and service delivery: a qualitative study. Int J Mental Health Promotion (2015) 17:5:261–77. 10.1080/14623730.2015.1080462

[29] Dean J Todd G Morrow H Mum, I used to be good looking….look at me now. The physical health needs of adults with mental health problems: the perspectives of users, carers, and front line staff. Int J Mental Health Promotion (2001) 4:16–24.

[30] Lloyd J Lloyd H Fitzpatrick R Peters M Treatment outcomes for schizophrenia qualitative research study of the view of family carers. BMC Psychiatry (2017) 17:266 10.1186/s12888-017-1418-8 28732482 PMC5521073

[31] Smith L Onwumere J Craig T Kuipers E. A role for poor sleep in determining distress in caregivers of individuals with early psychosis. Early Interv Psychiatry (2018). 10.1111/eip.12538 29417730

[32] Perlick DA Hohenstein JM Clarkin JF Kaczynski R Rosenheck RA. Use of mental health and primary care services by caregivers of patients with bipolar disorder: a preliminary study. Bipolar Disord. (2005) 7:126–35. 10.1111/j.1399-5618.2004.00172.x 15762853

[33] Poon AWC Curtis J Ward P Lonergan C Lappin J. Physical and psychological health of carers of young people with first episode psychosis. Australas Psychiatry (2018) 26:184–8. 10.1177/1039856217748250 29334229

[34] Gupta S Isherwood G Jones K Van Impe K. Assessing health status in informal schizophrenia caregivers compared with health status in non-caregivers and caregivers of other conditions. BMC Psychiatry (2015) 15:162. 10.1186/s12888-015-0547-1 26194890 PMC4509463

[35] NICE National Institute of Health and Clinical Excellence National Institute of Health and Clinical Excellence: Core Interventions in the Treatment and Management of Schizophrenia in Primary and Secondary Care. London: NICE (2014).

[36] National Health Service England Commissioning for Quality and Innovations (CQUIN) (2016). Available online at: https://www.england.nhs.uk/wp-content/uploads/2016/03/cquin-guidance-16-17-v3.pdf

[37] Mitchell AJ Delaffon V Vancampfort D Correll CU De Hert M. Guideline concordant monitoring of metabolic risk in people treated with antipsychotic medication: systematic review and meta-analysis of screening practices. Psychol Med. (2012) 42:125–47. 10.1017/S003329171100105X 21846426

[38] Chatzidamianos G Lobban F Jones S. A qualitative analysis of relatives', health professionals' and service users' views on the involvement in care of relatives in Bipolar Disorder. BMC Psychiatry (2015) 15:228. 10.1186/s12888-015-0611-x 26403843 PMC4582817

[39] Cree L Brooks H Berzins K Fraser C Lovell K Bee P. Carers' experiences of involvement in care planning: A qualitative exploration of the facilitators and barriers to engagement with mental health services. BMC Psychiatry (2015) 15:208. 10.1186/s12888-015-0590-y 26319602 PMC4553006

[40] Askey R Holmshaw J Gamble C Gray R What do carers of people with psychosis need from mental health services? Exploring the views of carers, service users and professionals. J Fam Ther. (2009) 31:310–31. 10.1111/j.1467-6427.2009.00470.x

[41] Onwumere J Kuipers E Caregiving roles: when will they be routinely recognised and supported? J Ment Health (2017) 26:95–7. 10.1080/09638237.2017.1301649 28355950

[42] Poon AWC Harvey C Mackinnon A Joubert LA Longitudinal population-based study of carers of people with psychosis. Epidemiol Psychiatr Sci. (2016) 5:1–11. 10.1017/S2045796015001195 PMC6998635 26847994

[43] Onwumere J Shiers D Chew Graham C Understanding the needs of carers in general practice. Br J Gen Pract. (2016). 66:400–1. 10.3399/bjgp16X686209 27481960 PMC4979915

